# The Cathepsin Family in Disease: From Molecular Mechanisms to Therapeutic Applications

**DOI:** 10.2174/011570159X382799250721072924

**Published:** 2025-08-08

**Authors:** Lorca Alzoubi, Yassmen Hamzat, Alaa Alqudah, Alaa A.A. Aljabali

**Affiliations:** 1 Department of Medicinal Chemistry and Pharmacognosy, Faculty of Pharmacy, Yarmouk University, P.O. Box 566, Irbid, 21163, Jordan;; 2 Department of Pharmaceutics and Pharmaceutical Technology, Faculty of Pharmacy, Yarmouk University, P.O. Box 566, Irbid, 21163, Jordan

**Keywords:** Cathepsins, proteolytic enzymes, therapeutic targeting, pathological conditions, drug development, molecular mechanisms

## Abstract

The cathepsin family of proteolytic enzymes is involved in the maintenance of major physiological processes, including protein degradation, immune modulation, tissue remodeling, and apoptosis. Members of the cathepsin family include cysteine, serine, and aspartic proteases, which are implicated in diverse cellular functions. Evidence for tissue-specific expression emphasizes the specialized functions of these enzymes in many organs. However, dysregulated cathepsin activity has been implicated in a wide range of pathological conditions, including, but not limited to, cancer, cardiovascular diseases, neurodegeneration, and autoimmune disorders. There is significant therapeutic potential for intervention, whereby specific inhibitors of certain cathepsins may offer promising strategies for disease management. Despite this promise, major challenges persist in designing inhibitors that avoid off-target effects while respecting the dual physiological and pathological roles of cathepsins. Structural similarities among family members and their context-dependent functions complicate precision targeting. This review identifies the emerging strategies including structure-guided design, cathepsin-cleavable delivery systems, and real-time imaging that are reshaping therapeutic approaches toward these complex enzymes. A structured web-based literature search was conducted using PubMed, Scopus, and Google Scholar employing keywords such as “cathepsins”, “therapeutic targeting”, “proteolytic enzymes”, and “disease pathways” to inform this review. As cathepsins continue to play a key role in health and disease, much research is warranted to determine their full therapeutic potential, which would represent a foundation for treatment options for various complex diseases.

## 1 INTRODUCTION

### 1.1 The Cathepsin Family Overview

The cathepsin family of proteases is significant in physiological processes and pathological conditions, and the research on their involvement in diseases continues to evolve [1]. This review investigates key studies related to the role of cathepsins in the pathogenesis of diseases, such as cancer, cardiovascular diseases, neurodegenerative diseases, and inherited disorders, with a focus on their potential as therapeutic targets. Recent studies have highlighted the complex interactions between cathepsins, the dual nature of their involvement in disease progression, and obstacles to their therapeutic targeting. The involvement of cathepsins in disease mechanisms is complex, as both supportive and conflicting evidence is present in various diseases [2-7]. In other words, the study of cathepsins in cancer, neurodegenerative diseases, cardiovascular diseases, and inborn errors has revealed their dual role as pathological agents and protective regulators, depending on the context [8]. The emerging concept of “cathepsin cannibalism” and the nuanced understanding of its role in diseases, such as cancer and cardiovascular diseases, underlines the fact that any future strategy will have to consider the complex interplay and pleiotropy of these proteases [9, 10]. Although classical approaches have targeted the inhibition of cathepsins to mitigate their pathogenic effects, emerging strategies emphasize selective modulation of their enzymatic activity to achieve more favorable outcomes. The ongoing debate within the field emphasizes that further research on cathepsins is necessary for greater precision in therapeutic intervention [11].

Cathepsins are a diverse family of lysosomal cysteine proteases that modulate several physiological and pathological processes. They mediate protein turnover, antigen presentation, invasion through the extracellular matrix, and immune response regulation [2]. The highly conserved structural and functional features of cathepsins across eukaryotic species underscore their evolutionary significance as regulators of intracellular proteostasis and immune adaptation, providing unique insights into the biology of cellular homeostasis [12].

### 1.2 Methodology

This review adopted a narrative synthesis approach to systematically explore the biological functions, pathological implications, and therapeutic potential of cathepsins. To ensure comprehensive coverage, a structured literature search was conducted across four major scientific databases: PubMed, Scopus, Web of Science, and Google Scholar. The search focused on peer-reviewed publications from 2000 to 2024, with particular attention to high-impact studies in the past decade. Keywords were used in various combinations, including: “cathepsins”, “lysosomal proteases”, “cysteine proteases”, “disease mechanisms”, “cathepsin inhibitors”, “extracellular matrix remodeling”, “neurodegeneration”, “autoimmune diseases”, and “drug targeting”.

The inclusion criteria encompassed original research, reviews, and meta-analyses that investigated the structural biology, molecular functions, or pathological roles of cathepsins in mammalian systems, particularly in human health and disease. Priority was given to studies offering mechanistic insights or translational relevance across various fields, including cancer biology, cardiovascular pathology, neurodegeneration, immune regulation, and infectious diseases.

Studies were excluded if they were non-English, non-peer-reviewed, methodologically insufficient, or outdated and superseded by newer findings. To provide a multidimensional perspective, literature related to inhibitor development, structural modeling, and targeted delivery strategies was deliberately integrated.

The final selection was organized thematically into categories: (1) cathepsin structure and classification, (2) physiological and disease-associated functions, (3) development of selective inhibitors, (4) therapeutic delivery mechanisms, and (5) clinical research frontiers. Emphasis was placed on recent advances supported by experimental data, structural biology, and novel imaging or pharmacological platforms. This methodological design ensured that the synthesis was current, comprehensive, and aligned with ongoing translational and clinical developments in cathepsin-related research.

### 1.3 Classifications and Evolutionary Relationships of Cathepsins

Based on their structural and functional differences, cathepsins have been further classified into several subtypes, including cathepsins B, L, K, S, C, and V [13, 14]. This classification reflects their substrate specificity, catalytic residues, and subcellular localization. Evolutionary conservation has underlined the importance of these enzymes in cellular processes, with specific subtypes evolving to satisfy the unique biological requirements of various organisms. Phylogenetic analyses reveal that several cathepsins have undergone adaptive divergence, resulting in tissue-specific roles, such as cathepsin K in osteoclast-mediated bone matrix degradation and cathepsin S in antigen processing within immune cells. As shown in Table **1**, these proteases are integral to protein catabolism, apoptosis regulation, antigen presentation, and extracellular matrix (ECM) remodeling. The spatial distribution of individual cathepsins across tissues directly influences their involvement in various disease processes, including tumor invasion, neurodegenerative disease progression, bone resorption disorders, and chronic autoimmunity. Targeted pharmacological strategies have been developed against these enzymes; for instance, selective inhibition of cathepsin K has shown efficacy in osteoporosis models, while cathepsin S inhibitors demonstrate therapeutic potential in immune-mediated pathologies.

### 1.4 Historical Discovery and Characterization of Cathepsins

Cathepsins were first identified in the late 19^th^ century based on their lysosomal proteolytic activity. Their biochemical properties and physiological relevance became more clearly defined during the second half of the 20^th^ century as advancements in protein chemistry enabled structural and functional isolation [15, 16]. Subsequent investigations established their central role in intracellular degradation pathways and expanded their functional repertoire to include regulatory roles in apoptosis, autophagy, and inflammation [17, 18].

### 1.5 General Biological Functions and Tissue Distribution Patterns

Cathepsins, primarily localized in lysosomes, can also be redistributed to other cellular compartments, depending on the extracellular regions under specific physiological or pathological conditions, for example, during tissue remodeling within the extracellular matrix. Beyond proteolysis, cathepsins participate in extracellular matrix turnover, modulation of immune responses, and intracellular signaling cascades [2, 19]. Their tissue-specific expression patterns reflect distinct biological roles. For example, cathepsin K is predominantly expressed in osteoclasts, where it facilitates bone matrix resorption, whereas cathepsin S is enriched in antigen-presenting cells, supporting its role in immune regulation [20, 21]. In pathological settings, cathepsins are implicated in the progression of cancers, cardiovascular disorders, and neurodegenerative diseases [19]. Conversely, they may also contribute to tissue repair and regeneration, reflecting their dual roles in both damage and recovery. These multifaceted activities make cathepsins compelling therapeutic targets, though their context-dependent effects continue to generate debate regarding the selectivity and timing of their pharmacological inhibition [22].

### 1.6 Evolutionary Significance and Roles of Cathepsins

Cathepsins are a family of proteolytic enzymes that have emerged as central mediators of both cellular and systemic physiology, reflecting their evolutionary conservation and adaptive functional expression [23]. While predominantly categorized as cysteine proteases, certain cathepsin family members also include serine and aspartyl variants. These enzymes are present across prokaryotic and eukaryotic domains, underscoring their fundamental biological importance. Their evolutionary trajectory illustrates a delicate balance between structural preservation and functional specialization, allowing integration into a diverse range of physiological and immunological processes [24, 25].

This highlights the indispensable functions of cathepsins in fundamental cellular processes, including protein degradation and turnover, which are vital for maintaining cellular homeostasis. Phylogenetic studies indicate that cysteine cathepsins, including cathepsins B, L, and K, originate from a common ancestral protease, all of which share conserved structural motifs and active site architectures that underscore their roles in lysosomal protein catabolism [26, 27]. However, evolutionary pressures have driven functional diversification, enabling some cathepsins to fulfill tissue-specific physiological requirements. For instance, cathepsin K has evolved a specialized function in bone matrix degradation, a continuous process essential for skeletal remodeling. In contrast, cathepsin S has adapted to perform antigen processing in professional antigen-presenting cells, a key function in adaptive immunity [28].

The functional repertoire of cathepsins extends beyond lysosomal protein degradation. These enzymes are critically involved in ECM remodeling through the proteolytic breakdown of structural degradation of collagen and elastin, a process predominantly mediated by cathepsins K and L [2]. This proteolytic activity contributes to various physiological processes, such as tissue repair, angiogenesis, and morphogenesis. Cathepsins also play a central role in modulating immune responses. For instance, cathepsins S and L facilitate antigen presentation by hydrolyzing invariant chain components within major histocompatibility complex class II (MHC II)-expressing antigen-presenting cells [29, 30]. In addition, cathepsins B and L participate in both apoptosis and autophagy, indicating their context-dependent roles in either promoting cell survival or executing programmed cell death [31, 32]. The cell-specific adaptation of cathepsin K to osteoclast-mediated bone matrix resorption further underscores the functional specialization of these enzymes in fulfilling tissue-targeted physiological roles [33].

Despite their essential physiological roles, dysregulated cathepsin activity has been implicated in a broad spectrum of human pathologies [34]. Overexpression or aberrant subcellular localization can result in the uncontrolled degradation of the extracellular matrix components, contributing to inflammatory disorders, such as rheumatoid arthritis and cardiovascular disease. Conversely, deficiencies in specific cathepsins, such as cathepsin K, are associated with severe skeletal phenotypes, including osteopetrosis, due to impaired bone resorption [35]. Comparative evolutionary analyses have further elucidated the functional divergence of cathepsins. In parasitic species, these enzymes have evolved to efficiently degrade host-derived proteins for nutrient acquisition, reflecting their ecological specialization [36]. In mammals, the tissue-restricted expression and immunoregulatory activity of certain cathepsins particularly in immune-privileged environments and skeletal systems highlight the influence of evolutionary pressures shaped by both environmental and physiological demands [37]. Cathepsins exemplify the evolutionary balance between conservation and innovation. They retain core proteolytic mechanisms while adapting to specialized, tissue-specific tasks, underscoring their dual relevance in homeostasis and disease [38]. Understanding their evolutionary trajectory provides not only an insight into their biological complexity, as illustrated in Fig. (**1**), but also a mechanistic foundation for therapeutic strategies targeting these enzymes across a range of pathological conditions [39].

A recent report introduced the concept of "cathepsin cannibalism", wherein cathepsins preferentially degrade one another rather than their classical extracellular matrix targets, such as collagen or elastin [40, 41]. This challenges the longstanding view that cathepsins function independently and non-interactively upon proteolytic substrates, suggesting instead a higher-order regulatory mechanism in which cathepsins modulate each other’s activity, influencing tissue remodeling dynamics [42]. This phenomenon has been experimentally validated through fluorogenic substrate assays and multiplex zymography [43]. These findings support a paradigm shift in how proteolytic regulation is conceptualized during tissue remodeling, with potential implications for disease progression. Understanding how cathepsin-cathepsin interactions influence pathophysiological states may inform the development of therapeutic strategies that more precisely target protease networks rather than individual enzymes [41, 44-46].

Cathepsins comprise a diverse group of proteases, each with distinct physiological and pathological functions, as summarized in Table **2**. These enzymes, classified according to their active-site residues, which include cysteine, aspartic acid, or serine, participate in essential biological processes, including lysosomal proteolysis, antigen presentation, apoptosis, and ECM remodeling. Their tissue-specific expression profiles contribute to the pathogenesis of a broad range of diseases, including cancer, neurodegenerative disorders, autoimmune conditions, and cardiovascular pathology. For example, cathepsin K is central to bone matrix resorption and has been targeted therapeutically for the treatment of osteoporosis. Cathepsin S, on the other hand, modulates immune responses and is implicated in the pathogenesis of chronic inflammation and autoimmunity. The therapeutic modulation of cathepsins presents distinct challenges, requiring selective inhibition that preserves physiological functions while minimizing off-target effects, as outlined in Table **2**.

## 2 SIGNIFICANCE IN HUMAN HEALTH

### 2.1 Overview of Disease-Relevant Mechanisms and Therapeutic Implications of Cathepsins

Cathepsins are diverse lysosomal proteases that play critical regulatory roles in a wide range of physiological and pathological processes [1, 5, 16, 20, 30]. Based on the identity of their active-site amino acid residues, these enzymes are grouped into cysteine proteases including cathepsins B, L, K, and S serine proteases, such as cathepsins A and G, and aspartic proteases including cathepsins D and E. Comparative evolutionary analyses highlight their conserved molecular architecture and core functions, which are maintained across species. These functions include protein degradation, antigen processing, and extracellular matrix remodeling [7, 10, 23]. The disease-specific involvement of cathepsin in neurodegenerative and cardiovascular pathologies is shown in Fig. (**2**).

### 2.2 Physiological Roles and Distribution

Cathepsins play a crucial role in cellular homeostasis by regulating intracellular protein turnover, hormone activation, and tissue remodeling [45]. Their expression patterns are highly tissue-specific; cathepsin K is predominantly expressed in bone tissues, where it facilitates osteoclast-mediated matrix degradation, while cathepsin S is enriched in immune cells, where it modulates antigen presentation and inflammatory signaling [20]. Dysregulation of these enzymes disrupts key physiological processes and contributes to the development of pathological conditions across multiple organ systems [12].

Dysregulation of cathepsin activity contributes to the pathogenesis of multiple disease states. In cardiovascular disorders, cathepsin S degrades elastin within atherosclerotic plaques, promoting local inflammation and destabilizing plaque architecture [47]. These effects are linked to aneurysm formation and endothelial dysfunction, both of which exacerbate vascular risk [47, 48]. In malignancies, cathepsins facilitate tumor progression through mechanisms such as extracellular matrix degradation, stimulation of angiogenesis, and modulation of immune escape [49]. Among these enzymes, cathepsin B is particularly associated with increased metastatic potential and is also involved in regulating programmed cell death. In neurodegenerative conditions, including Alzheimer’s disease, cathepsins contribute to neuroinflammation and pathological protein accumulation, notably the aggregation of tau and other misfolded proteins [50, 51].

### 2.3 Therapeutic Potential of Cathepsin Inhibitors

The therapeutic potential of cathepsin inhibitors stems from their capacity to selectively modulate pathological processes associated with inflammation, cardiovascular disease, and bone degradation. For instance, brensocatib, a dipeptidyl peptidase-1 inhibitor, targets cathepsin C and has been shown to reduce neutrophil serine protease activity in chronic inflammatory conditions, such as bronchiectasis [52-54]. LY3000328 is a selective inhibitor of cathepsin S, demonstrating efficacy in attenuating atherosclerotic plaque progression. In skeletal disorders, odanacatib inhibits cathepsin K, effectively reducing bone resorption and offering clinical benefits in the management of osteoporosis [55, 56].

However, the clinical application of cathepsin-targeted therapies remains limited by significant biological and pharmacological complexities. Many cathepsins exhibit overlapping substrate specificities and are co-expressed in multiple tissues, complicating efforts to achieve selective inhibition without disrupting physiological proteostasis. Additionally, their roles can shift depending on disease stage, cell type, or inflammatory context, requiring spatiotemporally controlled therapeutic strategies. Inhibitors must therefore be precisely tuned not only to target the correct protease, but also to preserve its context-dependent protective functions while limiting pathological activity. These challenges underscore the need for structure-guided inhibitor design, improved biomarker-driven patient stratification, and delivery systems capable of achieving compartment-specific modulation.

### 2.4 Challenges in Therapeutic Targeting

Despite their therapeutic potential, cathepsins remain difficult to target pharmacologically. Structural similarities among family members hinder the development of highly selective inhibitors, increasing the likelihood of off-target interactions [19]. Moreover, many cathepsins exhibit dual functionality serving both protective and pathogenic roles. Effective inhibition of disease-associated cathepsin activity often requires sustained, systemically delivered regimens that address enzymatic redundancy and tissue-specific compensatory mechanisms. For example, while inhibition of cathepsin S may reduce inflammatory signaling, it can also impair tissue repair processes and antigen presentation in immune-competent tissue [57, 58].

### 2.5 Emerging Applications and Future Directions

Beyond their established roles in protein degradation and immune regulation, cathepsins are gaining attention as novel therapeutic targets in oncology and neurodegeneration [45, 46, 54]. Their enzymatic contribution to tumor microenvironment remodeling suggests a role in modulating cathepsin activity, which may lead to a reduction in neurodegenerative disorders. Selective modulation of cathepsin activity may reduce neuroinflammation and promote neuronal survival, particularly in protein aggregation pathologies [8]. Recent advances in molecular modeling and high-throughput screening technologies have improved the precision and efficacy of cathepsin inhibitor development [27, 34, 50].

As central mediators at the interface of physiological and pathological processes, cathepsins remain a compelling focus of translational research [14, 18, 51]. Although challenges persist in achieving isoform specificity and minimizing off-target effects, preclinical and early clinical studies indicate therapeutic potential across inflammatory, cardiovascular, oncological, and neurological disorders. Future investigations must refine delivery strategies, enhance biomarker-guided patient selection, and address mechanistic gaps to translate these inhibitors into clinically viable treatments [27, 59].

### 2.6 Clinical Relevance of the Cathepsin Family

#### 2.6.1 Cardiovascular Diseases

Cathepsins are a major group of lysosomal proteases with functions spanning both physiological regulation and pathological disruption [60]. These enzymes contribute to cellular homeostasis, immune modulation, and tissue modeling, underpinning their relevance in health and disease [31]. Clinical consideration of the functions of these enzymes has highlighted their therapeutic potential for therapies, but has also faced obstacles to their modulation [61]. In cardiovascular pathology, cathepsins, particularly cathepsin S, play a prominent role in disease progression [62]. Cathepsin S facilitates elastin degradation within atherosclerotic plaques, driving inflammation, structural instability, and progression toward myocardial infarction and stroke. Elevated levels of cathepsin S correlate with increased cardiovascular risk, positioning it as a promising therapeutic target for plaque stabilization and inflammation control [63, 64].

#### 2.6.2 Cancer Biology

Other cathepsins, including L and K, contribute to vascular remodeling and have been implicated in the pathogenesis of aneurysm formation and hypertension [65, 66]. Preclinical studies indicate that selective inhibition of these enzymes may reduce plaque burden, enhance endothelial function, and stabilize vascular architecture, supporting their therapeutic potential in the management of cardiovascular diseases [67]. Among other processes, cathepsins play key roles in tumor development and are associated with extracellular matrix degradation, neoangiogenesis, and immune escape [57]. Cathepsin B, in particular, degrades basement membrane components, thereby facilitating metastasis and enhancing the invasiveness of cancer cells. Additionally, it modulates apoptotic and autophagic pathways, which are critical to tumor survival and adaptation [68]. Elevated cathepsin activity is frequently observed in aggressive tumor phenotypes and is correlated with poor clinical outcomes. These oncogenic effects are further amplified by cathepsins L and D, which support tumor invasiveness and confer resistance to chemotherapy, making them high-priority targets in anticancer drug development [69, 70]. Experimental inhibitors targeting cathepsins B and L have shown encouraging results in preclinical cancer models, demonstrating their ability to suppress tumor growth and reduce metastatic dissemination [71, 72].

#### 2.6.3 Neurodegenerative Diseases

Cathepsins have been shown to serve both beneficial and harmful roles in the nervous system. In healthy tissue, they support protein turnover and immune function. However, when dysregulated, they can worsen neuroinflammation and contribute to neuronal loss and toxic protein accumulation [50]. One example is cathepsin B, which may help clear amyloid-beta in Alzheimer’s disease but has also been linked to neuronal damage when overactive. Similarly, deficiencies in cathepsin L have been associated with tau buildup, a known contributor to disease progression [33, 42, 61, 68, 72]. Because these enzymes behave differently depending on the cellular and disease context, therapeutic strategies involving cathepsin modulation remain difficult to design. Nonetheless, some recent findings suggest that fine-tuning their activity rather than full inhibition might offer a way to reduce harmful inflammation while supporting neuron survival [50].

#### 2.6.4 Inflammatory and Autoimmune Conditions

Cathepsins are centrally involved in regulating immune balance and inflammatory signaling. In particular, cathepsin C plays a role in neutrophil activation and has been linked to airway and joint inflammation, as seen in bronchiectasis, chronic obstructive pulmonary disease (COPD), and rheumatoid arthritis [16, 23]. Clinical interest has focused on brensocatib, a compound that selectively inhibits cathepsin C and has shown measurable reductions in neutrophilic inflammation among patients with bronchiectasis, with corresponding improvements in clinical endpoints [73-75]. Another relevant protease is cathepsin S, which supports antigen processing and MHC class II presentation. Its heightened activity has been observed in autoimmune disorders, such as systemic lupus erythematosus and multiple sclerosis [76]. Preclinical studies suggest that targeting cathepsin S may help modulate autoimmune responses by reducing antigen-driven T-cell activation, opening new avenues for treatment development [77].

#### 2.6.5 Bone Metabolism and Skeletal Disorders

Cathepsin K is predominantly expressed in osteoclasts and plays a central role in degrading type I collagen during bone matrix resorption. Its overactivity has been linked to skeletal disorders, such as osteoporosis and bone metastasis, where excessive bone turnover disrupts structural integrity [20, 78]. Therapeutically, odanacatib has been developed as a selective inhibitor of cathepsin K. Clinical trials have shown that it effectively reduced bone resorption while sparing bone formation, an advantage over earlier antiresorptive agents [79]. Nonetheless, further development was halted due to emerging concerns about cardiovascular risk, raising caution about the need for precise molecular targeting and long-term safety profiling in cathepsin-based therapies [80, 81].

#### 2.6.6 Challenges in Targeting Cathepsins

Despite clear therapeutic potential, targeting cathepsins presents unique challenges due to overlapping substrate specificities, off-target toxicity, and compensatory upregulation.

These challenges are compounded by the context-dependent roles cathepsins play in various diseases sometimes pathogenic, sometimes protective making selective inhibition highly complex. Additionally, cathepsins often exhibit redundancy across isoforms, which can reduce therapeutic impact if not all relevant enzymes are inhibited simultaneously.

### 2.7 New Emerging Roles of Infectious Diseases

Cathepsins have been increasingly recognized for their contributions to host-pathogen interactions, particularly in facilitating viral and parasitic entry. For instance, cathepsin L cleaves and activates viral fusion proteins, enabling the entry of certain viruses into host cells. This role makes it a viable target for antiviral strategies [59, 82]. In parasitic infections, cathepsins play a crucial role in the degradation of host proteins, thereby assisting parasite survival and immune evasion, reinforcing their relevance in the pathobiology of infectious diseases.

Despite the therapeutic promise of cathepsin inhibition, several obstacles remain. High structural similarity among cathepsin isoforms complicates the development of selective inhibitors and increases the potential for off-target effects [83]. Their dual role in physiology and pathology further adds to the complexity; inhibiting a cathepsin involved in inflammation might inadvertently disrupt essential processes, such as antigen presentation or tissue remodeling. For example, blocking cathepsin S may reduce inflammatory signaling but impair MHC class II antigen processing and delay tissue repair [57]. Recent advances in molecular modeling and high-throughput compound screening have contributed to the identification of next-generation inhibitors with improved specificity and potency. Emerging strategies also include combination regimens either involving multiple cathepsin inhibitors or combining them with agents that target complementary pathways which may improve therapeutic outcomes and reduce resistance [84]. The cathepsin family comprises a versatile group of proteases with established roles across oncology, neurodegeneration, cardiovascular disease, and infectious pathology. Continued progress in understanding their biochemical behavior and context-specific actions is refining our approach to cathepsin-targeted therapy. As development efforts progress, the critical challenge will be achieving a therapeutic balance maximizing disease control while minimizing disruption to protective functions essential for physiological homeostasis [18, 51, 59].

### 2.8 Research Gaps and Significance

Despite advances in molecular biology and proteomics, cathepsins remain under-characterized as a functionally interconnected family. These proteases participate in numerous biological systems; however, many of their shared regulatory mechanisms and context-dependent roles remain poorly understood. A significant limitation in the current literature is its fragmentation; most studies focus on individual cathepsin isoforms or disease-specific settings, leaving broader physiological interactions poorly defined. This piecemeal approach has hindered efforts to construct a unified model explaining how cathepsins contribute to both tissue maintenance and pathology. A more systematic framework is needed one that accounts for isoform crosstalk, tissue-specific regulation, and compensatory activity across disease states. Addressing these gaps will be critical for advancing cathepsin-targeted interventions and clarifying their full therapeutic potential.

This comprehensive review is, therefore, necessary because there are three major research gaps in the field. First, the molecular aspects of cathepsin far exceed the traditional categorical understanding of these enzymes. Indeed, emerging evidence suggests that the activities of these proteases extend beyond simple degradative roles but function as sophisticated molecular switches with context-dependent regulatory competence. However, no inclusive synthesis that truly maps these multifaceted interrelations across diverse biological systems at all levels is available.

Secondly, technological advances in molecular imaging, computational biology, and system-wide profiling have generated unprecedented datasets that remain insufficiently integrated. The convergence of high-resolution structural analysis, activity-based probing, and advanced computational modeling presents an opportunity to reframe our understanding of cathepsin functionality; however, this potential remains largely unrealized.

Third, the therapeutic landscape of cathepsin represents a complex frontier with immense potential but is also fraught with daunting challenges. Current paradigms of inhibition have resulted in little translational success, and thus, there is a need for scientific research to revisit our fundamental knowledge of the mechanisms behind these enzymes, which is somewhat fundamentally incomplete. Moreover, it provides a systematic review that indicates clear molecular targeting strategies against diseases involving cathepsin activity can be identified beyond the somewhat limited traditional paradigms of inhibition.

The study of cathepsins spans multiple disciplines, demanding integration across molecular biology, clinical research, and therapeutic development. This review assesses the current landscape of knowledge, highlights critical areas where understanding remains limited, and outlines conceptual directions that may help reframe how the cathepsin family is approached in both experimental and clinical contexts.

### 2.9 The Unique Position of Investigation in Addressing these Critical Gaps is as Follows

More molecular mechanisms of cathepsin need to be explored on a pan-disciplinary and comprehensive scale across a range of different biological systems and organ types to achieve a better understanding. The application of various emerging technologies, high-resolution imaging, advanced proteomics, and advances in computational modeling has systematically explored the multifarious roles of cathepsins in diseases, from cancer to neurodegeneration, and beyond. These technologies now enable the identification of specific molecular targets for future therapeutic interventions, thus allowing for more precise treatments to modulate cathepsin activity rather than complete inhibition. Moreover, establishing a conceptual framework that resolves the existing interpretative conflicts regarding cathepsin functions will help integrate diverse findings and, consequently, facilitate the development of a clear understanding of their regulatory functions, thereby improving therapeutic strategies. This integrative approach will further refine existing models, improve clinical outcomes, and, in the process, drive the development of more effective cathepsin-targeted therapies.

The scientific community has reached an unparalleled juncture in technology capabilities, insight accumulated at the molecular level, and clinical imperatives, all of which point toward a cogent future. This review will be more than an exercise in retrospection and will have the intent and value of a strategic roadmap for further research. Such nuances, from an in-depth perspective, shall be afforded by the cathepsin family.

## 3 MOLECULAR BIOLOGY OF CATHEPSINS

Cathepsins are a group of proteolytic enzymes implicated not only in physiological processes but also in a wide range of pathological processes [6, 27, 67]. The molecular biology of cathepsins brings several structural intricacies, mechanisms of activation, regulatory pathways, and substrate specificity-all overarching principles that have shaped the broad biological functions and therapeutic potential of cathepsins [2, 46].

### 3.1 Structural Analysis

These enzymes catalyze the hydrolysis of peptide bonds in proteins and have been divided, based on the amino acid residues in their active sites, into cysteine, serine, and aspartic cathepsins, representing the three main families of proteases [15, 41, 50]. Members of the cathepsin family have been implicated in various physiological and pathological processes, including protein degradation, antigen presentation, and tissue remodeling. Despite their diverse functions, the structural features of cathepsins are well conserved within each family, which can shed light on their mechanisms of action and involvement in various diseases [23].

### 3.2 Cysteine Cathepsins: Papain-like Fold Structure

Cysteine cathepsins, including cathepsins B, L, and K, are papain-like lysosomal proteases [25]. This structural motif consists of two domains that together form a substrate-binding groove, within which the catalytic cysteine residue is positioned. In the active site of cysteine cathepsins, there is a catalytic dyad comprising a cysteine residue and a histidine residue, which are important for the proteolytic activity of these enzymes [85, 86]. For example, human cathepsin S has critical structural similarity to other cysteine proteases, such as papain and cathepsin K and L, based on the conservation of residues that are important for the active site of the enzyme [87]. The three-dimensional structure of the enzyme is described at a 2.5 Å resolution by X-ray crystallography, providing insight into the enzyme’s catalysis mechanism [88, 89].

Despite these conserved structural features, cysteine cathepsins exhibit functional diversity. Although cathepsin L has emerged as a key lysosomal processing protease, recent evidence implicates it in the degradation of histones, and its role appears to extend well beyond the classic mission of protein degradation.

### 3.3 Serine and Aspartic Cathepsins - Divergent Structural Features

Cysteine cathepsins adopt a papain-like fold, whereas serine cathepsins adopt a chymotrypsin-like fold, which is composed of two six-stranded β-barrel domains [2, 26]. Serine cathepsin, on the other hand, contains a catalytic triad including serine, histidine, and aspartic acid residues involved in the hydrolysis of the peptide bond. The best examples are cathepsin A and cathepsin G, which possess a chymotrypsin-like fold [90-92]. In contrast, aspartic cathepsins adopt a biological structure in which each lobe contains a β-sheet surrounded by α-helices. Two aspartic acid residues in the cleft between the lobes form an active site that enables catalytic activity [45].

### 3.4 Functional Diversity and Disease Implications

The structural similarities of cysteine cathepsins underlie the considerable functional diversity of this enzyme family [61, 71]. For example, cathepsin B has been implicated in tumor progression and angiogenesis, whereas cathepsin S is involved in tumor invasion and immune modulation. At the same time, these studies suggest that the relationships between cathepsins will affect their involvement in the disease process [31, 42]. For example, cathepsin B degrades cathepsin S, thereby modulating its activity under pathological conditions [41].

Structural insights into cysteine cathepsins provide a better understanding of their role in diseases, including Alzheimer's, where changes in protease activity may modulate amyloid-beta peptide processing [51, 93]. In contrast, Cathepsin K was found to play a key role in bone resorption and, when deficient, it causes osteopetrosis; hence, this enzyme seems to be involved in bone homeostasis [94]. Generally speaking, cysteine cathepsins represent a papain-like fold that is conserved, but each family contains structurally and mechanistically specialized features. This is the structural basis of functional diversity [25, 85]. However, further structural analysis of cathepsins, along with their interactions in disease contexts, is necessary to elucidate their roles in health and disease completely and to inform the development of targeted therapeutic strategies for modulating their activity in disease conditions [39, 59].

### 3.5 Active Site Architecture

Cathepsins exhibit differences in their catalytic modes because the structural features of each active site determine the corresponding proteolytic activity of the enzymes [5, 38, 41]. In all cysteine cathepsins, such as cathepsins B, L, and K, an active-site cysteine and a histidine residue constitute a catalytic dyad, providing a locally chemically active environment that is efficient for nucleophilic attack on peptide bonds [50, 95]. In contrast, aspartic cathepsins utilize two aspartic acid residues within their active sites in a synergistic manner to hydrolyze the peptide bonds. Serine cathepsins, represented by cathepsins A and G, possess the characteristic serine, histidine, and aspartate catalytic triad, catalyzing proteolytic activity *via* a similar nucleophilic mechanism [61, 90]. These active sites have enabled the fine-tuning of substrate specificity and ensured the precise cleavage of target peptide bonds in even the most varied physiological and pathological contexts [41, 45, 62].

The activity of cathepsins is strictly regulated at several structural levels to ensure that their proteolytic function occurs appropriately in time and location [96, 97]. Most cathepsins are synthesized as inactive precursors or zymogens, which contain an inhibiting propeptide domain that prevents premature activation of their active sites. This domain should be cleaved for enzyme activation for proteolysis to occur only under the appropriate conditions [96].

For instance, cathepsin K contains a collagen-binding domain that allows its collagen-degrading activity to be specifically executed during bone resorption, a critical process in bone remodeling [97, 98]. In addition, these regulatory domains contribute to cellular localization and interactions with natural inhibitors, which control the activity of cathepsins under physiological conditions. These regulatory mechanisms are important for maintaining cellular homeostasis and preventing aberrant proteolysis, which may lead to pathological conditions [99, 100].

### 3.6 Structural-Function Relationships

Such a relationship between structure and function is apparent in the specialized activities of cathepsins [96]. For example, cathepsin K has an extended substrate-binding cleft that allows the enzyme to accommodate and degrade collagen, a key function in bone resorption. This structural adaptation is crucial for the role of cathepsin K in osteoclast-mediated bone degradation [20, 78, 94].

In contrast, cathepsin S is optimized for activity in mildly acidic environments of endosomes and lysosomes. Such activity is crucial for its role in antigen processing [30, 56, 87]. These structural features provide the basis for fine-tuning each cathepsin to a specific physiological role. This knowledge will prove helpful in the development of selective inhibitors for modulating cathepsin activity in diseases such as cancer, osteoporosis, and neurodegenerative disorders [9, 47, 48].

### 3.7 Structural Changes and Their Effects in Pathology

Structural changes in cathepsins may greatly alter their proteolytic activity, substrate specificity, and interaction with inhibitors [85]. Such alterations can determine the role of cathepsins in the disease pathology. Indeed, the highly active site-sensitive nature of mutations or conformational changes can cause either enhancement or inhibition of the activities of cathepsins [38, 101]. Such point mutations of important residues may reduce the ability of an enzyme to cleave peptide bonds; for example, cysteine or histidine residues in cysteine cathepsins have been associated with several diseases, including neurodegeneration and tumor progression [102, 103]. Structural conformational changes of cathepsin B in Alzheimer's disease have been linked with amyloid plaque accumulation, suggesting that the enzyme is unable to perform its normal activity in the brain [104].

In addition, regulatory domains, such as the propeptide domain, are highly important for regulating the timing and location of cathepsin activity [96]. Mutations or alterations in such domains may lead to premature activation, as in diseases such as rheumatoid arthritis or atherosclerosis, where excessive proteolysis of ECM components contributes to tissue damage and disease progression [105]. On the other hand, overactive inhibitors block necessary cathepsin functions and impede important processes, such as immune response or wound healing [98].

Changes in substrate specificity due to structural changes may also enable cathepsins to cleave nonphysiological substrates in a pathological manner [106]. For example, cathepsin L can cleave histones and other nuclear proteins, and changes in its substrate-binding site may affect cell cycle regulation or apoptosis. They may be implicated in diseases such as cancer or autoimmune disorders [107]. Such structural changes may lead to changes in cellular localization, as seen when cathepsins leak from lysosomes into the cytoplasm or extracellular space, a phenomenon that occurs in several diseases, including neurodegenerative diseases [108]. Leakage can precipitate apoptosis or necrosis, further exacerbating tissue damage. In tumor development, aberrant secretion into the extracellular matrix has been linked to increased invasive and angiogenic abilities, as cathepsins degrade components of the ECM [109].

These include active sites, regulatory domains, and substrate-binding clefts, which are important features in the structure-function relationship and are critical for the precise and regulated activity of cathepsins [110]. Alterations in these domains substantially affect the function of cathepsins, resulting in a wide array of pathological conditions [19]. Understanding how these structural changes affect cathepsin activity will thus be important in developing specific therapies aimed at modulating cathepsin function in diseases such as cancer, neurodegeneration, and cardiovascular disorders. Further investigation into these structure-function relationships will provide insights into therapeutic interventions and enhance our ability to exploit cathepsins as potential drug targets [111].

## 4 ACTIVATION AND REGULATION

### 4.1 Zymogen Processing

Because of this mistranslation or unregulated proteolysis, cathepsin proteolytic enzymes are synthesized as inactive precursors called zymogens [112]. Activation occurs through the cleavage of the propeptide domain, resulting in the exposure of the active site. This process is generally mediated by other proteases or autocatalytic reactions under specific conditions. For example, the movement of cathepsin D in lysosomes is autocatalytically activated in an acidic environment, where the pH allows for conformational changes that lead to enzyme activity [113].

### 4.2 pH-Dependent Activation

Cathepsin proteolytic activity is regulated by the acidic milieu of the lysosomes and endosomes. The optimum pH, which lies in the range of 4.5-5.5, is where all active site residues are protonated, thereby enhancing catalytic efficiency [113]. Conversely, at neutral pH, cathepsins exhibit low activity, resulting in minimal unwanted extracellular proteolysis. This pH-dependent regulation ensures the spatial and temporal control of proteolytic activity [2].

### 4.3 Cellular Compartmentalization

Cathepsin activity is tightly regulated by its subcellular distribution, which defines the spatial context of its functions. Most cathepsins are localized to lysosomes, where they participate in protein degradation and antigen processing under physiological conditions [114]. However, under pathological states such as cancer or chronic inflammation, certain cathepsins are secreted into the extracellular environment, where they contribute to matrix remodeling and disease progression [115]. This spatial redistribution highlights an additional layer of regulatory control through cellular compartmentalization.

### 4.4 Natural Inhibitors

Cathepsin activity is regulated by a set of endogenous inhibitors, including cystatins, serpins, and macroglobulins, which serve to limit proteolytic damage under physiological conditions [19]. Among these, cystatins specifically inhibit cysteine cathepsins by binding to their active sites, thereby blocking substrate entry and enzyme activation. Maintaining a functional balance between cathepsins and their inhibitors is essential for cellular homeostasis, and disruption of this balance has been linked to several pathological states [116].

### 4.5 Substrate Specificity

#### 4.5.1 Comparative Substrate Preferences

Cathepsins demonstrate distinct substrate specificities that align with their physiological roles. For instance, cathepsin K, which selectively cleaves collagen, is essential for osteoclastic bone resorption. In contrast, cathepsin S, active in antigen-presenting cells, processes invariant chain peptides involved in MHC class II antigen presentation [94, 117]. Aspartic cathepsins, such as cathepsin D, preferentially cleave peptide bonds adjacent to hydrophobic residues and contribute to intracellular protein turnover and hormone maturation [118].

#### 4.5.2 Specificity Determinants

The substrate specificity of cathepsins is governed by their active site configuration and the surrounding substrate-binding pockets [119]. Key residues within these binding grooves interact with the side chains of peptide substrates, guiding precise cleavage [120]. For example, the S2 subsite of cathepsin K accommodates proline-rich motifs, aligning with its role in collagen degradation. Mutations or conformational changes in these subsites can alter enzymatic specificity and affect catalytic efficiency [121].

Although cathepsins often exhibit distinct substrate preferences, considerable overlap in specificities is a common feature and often gives rise to functional redundancy or cross-reactivity [2]. This is especially evident in inflammatory conditions, where multiple cathepsins degrade extracellular matrix components, thereby contributing to tissue destruction [2]. The lack of strict exclusivity in substrate recognition complicates the development of highly selective inhibitors, as unintended off-target effects may reduce therapeutic precision.

Biological Implications: These specificity features carry major physiological and pathological consequences. For instance, excessive collagen degradation by overactive cathepsin K leads to osteoporotic bone loss. In contrast, the broad proteolytic activity of cathepsin B promotes tumor invasion and metastasis by remodelling the extracellular matrix [122-124]. Conversely, therapeutic modulation of specificity has clinical benefits: inhibitors targeting cathepsin S have shown efficacy in reducing inflammation associated with autoimmune disorders [125]. At the molecular level, cathepsins represent a structurally diverse group of enzymes whose regulation, activation, and substrate selectivity are tightly coordinated to ensure proper physiological function. When dysregulated, these same properties contribute to a wide spectrum of disease processes [126]. Understanding these structural and functional relationships is key to developing targeted therapies, although achieving selectivity remains a significant challenge. Future research should continue to clarify isoform-specific roles and translate these insights into novel treatment strategies [59].

## 5 PHYSIOLOGICAL FUNCTIONS

### 5.1 Physiological Functions of Cathepsins

Proteolytic cathepsins play an important role in a wide variety of physiological functions [16]. Besides their seemingly simple protein-degrading activity, the enzymes take part in cellular homeostasis, maturation of proteins, immune response, and tissue remodeling, to name a few examples [16]. Their proper regulation is critical for health, and their dysregulation contributes to a wide range of disease states. The subsequent sections provide a detailed discussion of the physiological functions of the cathepsins in more detail [23].

### 5.2 Processing of Proteins

#### 5.2.1 Protein Maturation

Generally, cathepsins participate in protein maturation and activation through their proteolytic activity, which converts inactive precursors into functional proteins [20, 80]. For instance, cathepsin K converts pro-collagen into collagen, thereby maintaining the integrity of bones and connective tissue [60, 99]. In addition, cathepsin L participates in the processing of histones, thereby facilitating proper chromatin remodeling [78, 94]. Moreover, cathepsin C is involved in the activation of granule-associated proteases, including neutrophil elastase, which is involved in innate immunity [54, 59].

Among others, cathepsin S, L, and B are involved in antigen presentation by MHC class II molecules, which is critical to the immune system. Cathepsin S, L, and B degrade the invariant chain; thus, these enzymes can bind antigenic peptides to activate CD4^+^ T cells. This is a critical event in adaptive immunity. Dysregulation of antigen processing has been implicated in impaired immune surveillance and the development of autoimmune diseases [13, 90].

The functions of cathepsins include the activation of peptide hormones, as evidenced by their ability to cleave specific precursors. Cathepsin D activates prolactin and other growth factors that are important for endocrine signaling [118]. B-Cathepsin also activates pro-insulin concerning glucose metabolism. Dysregulation of the enzymes involved in cathepsin hormone activation contributes to metabolic disorders and certain types of cancers [33, 61, 68, 72, 115].

Cathepsins play a vital role in ECM remodeling, particularly cathepsin K, which degrades type I collagen during bone resorption [35]. This is particularly important for different biological events, such as skeletal maintenance, wound healing, and tissue remodeling. Indeed, dysregulation may result in pathological conditions such as fibrosis, osteoporosis, or tumor invasion, as excessive degradation of the ECM supports metastasis [35].

#### 5.2.2 Cellular Processes

Cathepsins B and D cooperate in lysosomal clearance of autophagic cargo; loss of either enzyme impairs autophagy and accelerates aggregate build‑up in Alzheimer’s and Parkinson’s disease. Defective cathepsins lead to dysfunctional autophagy, promoting toxic aggregate accumulation in diseases such as Alzheimer's and Parkinson's [32].

Cathepsins induce apoptosis due to lysosomal membrane permeabilization. This results in the release of enzymes, such as cathepsin B and D, into the cytosol, leading to the activation of pro-apoptotic proteins, including Bid, and thus initiating caspase cascades [72, 118]. This lysosome-mediated pathway is utilized for the removal of virus-infected or damaged cells. On the other hand, the high activity of cathepsins increases the pathological conditions in diseases like ischemia-reperfusion injury [102, 109]. The involvement of cathepsins in apoptotic pathways is shown in Fig. (**3**).

More recently, active participation of cathepsins in cell signaling has also been demonstrated. For instance, cathepsin G exerts a complementary function in regulating inflammatory responses by activating protease-activated receptors, PARs [90-92]. Furthermore, cathepsins modulate the activity of different signaling intermediates, including cytokines and chemokines, and thereby regulating cell proliferation, differentiation, and immune activation [116, 126].

By processing antigens and modulating Toll‑like‑receptor signalling, cathepsins orchestrate both innate and adaptive immune responses. In addition to antigen processing, cathepsins such as S activate TLRs, enhancing pathogen recognition and immune activation [77]. Besides, they resolve inflammation by degrading pro-inflammatory mediators. However, the dysregulated activity of cathepsin increases illnesses associated with chronic inflammation, including rheumatoid arthritis and atherosclerosis [48, 63]. The functions of cathepsins are indispensable in a wide range of physiological processes, including protein maturation, antigen presentation, and cellular signaling [86, 122]. Their functions in autophagy, apoptosis, and immune responses further extend their crucial role in cellular and systemic homeostasis. While dysregulation of cathepsins has been implicated in several diseases, their strict regulation also opens possibilities for therapeutic intervention. Continuous research into their molecular mechanisms could enable the development of new treatments to target these versatile enzymes [32, 114].

## 6 PATHOLOGICAL ROLES IN MAJOR DISEASES

### 6.1 Pathologies of Cathepsins in Major Diseases

Cathepsins constitute a diverse group of proteolytic enzymes that play essential roles in maintaining physiological homeostasis [18]. However, when dysregulated, they contribute to a range of pathological processes, making them important targets in disease-specific therapeutic research [127]. This section will discuss their participation in cardiovascular diseases, cancer, inflammatory disorders, and bone conditions, pointing out specific mechanisms and relevant examples.

#### 6.1.1 Cardiovascular Diseases

Cathepsins contribute to cardiovascular pathology through their involvement in ECM degradation, promotion of inflammatory signaling, and modulation of vascular remodeling [3, 4, 61, 62].

##### 6.1.1.1 Atherosclerosis

Cathepsins S, K, and L degrade key structural components such as elastin and collagen, contributing to the weakening of the fibrous cap in atherosclerotic plaques. This degradation promotes plaque instability and increases the risk of rupture [30, 56]. Elevated cathepsin S activity has been associated with cytokine-driven inflammation which accelerates plaque progression. For instance, studies of patients with myocardial infarction have shown that unstable plaques express significantly higher levels of cathepsin S [64].

##### 6.1.1.2 Heart Failure

Cathepsins contribute to adverse cardiac remodeling by degrading ECM proteins and promoting cardiomyocyte apoptosis [35]. Among these, cathepsin B has been specifically implicated in post-infarction fibrosis and reduced ventricular function. Experimental knockout models lacking cathepsin B demonstrated improved cardiac outcomes, suggesting that therapeutic targeting of this protease may offer clinical benefit in the context of heart failure [42, 72].

##### 6.1.1.3 Angiogenesis

Cathepsins K and L contribute to angiogenesis by promoting endothelial cell migration and remodeling of the ECM. This enzymatic activity facilitates the release of pro-angiogenic mediators, including vascular endothelial growth factor (VEGF). However, when cathepsin activity is excessively upregulated, it may drive pathological angiogenesis, as observed in disorders such as diabetic retinopathy [1, 2].

##### 6.1.1.4 Vascular Remodeling

Dysregulated cathepsin activity promotes smooth muscle cell migration and ECM degradation, both of which contribute to pathological vascular remodeling [12]. In particular, cathepsins B and L have been implicated in aneurysm formation through their roles in weakening the arterial wall structure [61].

## 7 THERAPEUTIC DEVELOPMENT

### 7.1 Cathepsin-Targeting Therapeutic Development

With cathepsins being implicated in a wide array of pathological processes, they become an attractive target for therapeutic intervention [51, 57, 87, 124]. Significant effort is indeed put into the development of potent and safe cathepsin inhibitors [56, 103]. The development of cathepsin-targeted inhibitors has provided promising avenues for the therapy of diseases such as cancer, osteoporosis, and chronic inflammatory conditions [12, 57, 87, 91, 124]. Examples of relevant inhibitors, their therapeutic target, challenges, and phases of clinical development are summarized in Table **3**.

### 7.2 Drug Design Strategies

Recent advances in drug design strategies have made possible the preparation of highly potent and selective cathepsin inhibitors [75, 80, 98, 103, 107]. Structure-based approaches rely on high-resolution imaging techniques, such as X-ray crystallography and NMR spectroscopy, which give immense information on the three-dimensional structural architecture of the cathepsins [103]. Based on this knowledge, inhibitors can be designed to stereospecifically fit into the active site, thereby inhibiting enzymatic activity [15]. Another effective strategy pursued is fragment-based drug discovery. This involves the screening of libraries of small fragments that bind to the target protein with weak affinity. These fragments then undergo optimization or linking in efforts to generate potent inhibitors with enhanced selectivity [128].

Rational drug design has become, and will continue to be, an important contributor by integrating computational modeling with medicinal chemistry in the design of molecules that mimic the natural substrates or transition states of cathepsins [98]. Recent innovations include the *de novo* design of drugs and machine learning-assisted optimization which accelerate the identification of lead compounds with favorable pharmacokinetic and pharmacodynamic properties [129]. Novel targeting approaches, such as allosteric inhibition, have also been pursued to modulate cathepsin activity without directly competing with substrates at the active site. This allows for enhanced specificity and reduced off-target effects, one of the key challenges in therapeutic development [93].

### 7.3 Currently Represented Classes of Inhibitors

Different approaches to the development of cathepsin inhibitors have generated several distinct classes, each with its mechanisms of action and therapeutic potential [56]. Small-molecule inhibitors remain a prime focus because they can selectively target the active or allosteric sites of cathepsins. Recent examples include odanacatib, a cathepsin K inhibitor developed for the treatment of osteoporosis, and nitroxoline, originally an antibiotic that has been repurposed due to its potent cathepsin B inhibitory activity [56]. Inhibitors often attain high affinity and specificity by mimicking the transition states of enzymatic reactions [24, 130].

Peptide-based inhibitors have also received much interest [131]. By capitalizing on the natural substrate specificity of cathepsins, these peptides can be engineered for improved stability and cellular uptake. Recent developments include the design of cyclic peptides and peptidomimetics with improved pharmacokinetic properties [116]. Antibody-based approaches provide a highly attractive strategy, especially toward cathepsins whose expression is induced in a specific pathological setting. Monoclonal antibodies and antibody-drug conjugates have indeed shown high specificity and potential for targeted delivery into tumors [132].

Many natural products derived from plants and microorganisms have yielded numerous inhibitors of cathepsins [133, 134]. Most of those are very original in their mechanisms and thus provide lead scaffolds for eventual synthetic optimization. Several natural products have been reported to exhibit inhibitory activities against cathepsins, which may be promising lead compounds in drug development [55].

### 7.4 Clinical Development Status

Success and pitfalls alike characterize the clinical development of cathepsin inhibitors. One notable success is the odanacatib development program, in which dramatic efficacy was demonstrated in bone mineral density increments in osteoporosis patients [79-81]. Although the program was later withdrawn from clinical development due to cardiovascular safety concerns, it was one of the first programs testifying to the therapeutic potential of targeting cathepsin K for the treatment of bone-related disorders. However, several of these have encountered issues. The early-stage trials of the inhibitors of cathepsin B, for instance, were discontinued because of specificity and off-target effects [81].

Several clinical trials are currently in progress for the repositioning of these inhibitors in various cancers, inflammatory diseases, and bone disorders [135]. The newer candidates, including inhibitors of cathepsin S, demonstrate beneficial activity against neuropathic pain and autoimmune conditions. Ongoing studies are expected to result in a clear determination of the safety profile and therapeutic efficacy in well-defined patient populations [129, 133].

Safety remains one of the top concerns in the development of cathepsin inhibitors. The structural similarity of cysteine proteases poses a challenge to selectivity. Inhibition of off-target proteins has the potential to give rise to adverse effects [38, 136]. Thus, an appropriate therapeutic window must be established, where potent inhibition is balanced against an acceptable safety profile. Approaches such as localized drug delivery and transient inhibition are being explored to minimize systemic toxicity while escalating therapeutic efficacy [13]. The cathepsins represent a very promising target for therapies treating various pathological conditions. A set of advances in drug design strategies, such as structure-based approaches, fragment-based discovery, and rational design, has notably helped in the development of selective and potent inhibitors. Specificity and safety concerns make their translation into clinical practice challenging; however, these are continuously being refined in ongoing research. Overcoming these challenges may enable cathepsin inhibitors to emerge as game-changing therapies for a wide range of diseases. Fig. (**4**) highlights cathepsin-mediated processes in inflammatory and autoimmune diseases.

## 8 EMERGING TECHNOLOGIES AND APPROACHES

### 8.1 Emerging Technologies and Methods

The development of new technologies has revolutionized our understanding, diagnosis, and therapies for complex diseases. New methods and therapeutic approaches are opening promising pathways toward improvements in diagnostic precision and therapeutic efficacy [137, 138]. This section describes recent advances in the state of the art in detection technologies and therapeutic approaches, underlining their potential to address unmet medical needs.

### 8.2 Cathepsin‑Targeted Imaging and Diagnostic Tools

Although many advanced detection tools have yet to be applied directly to cathepsins, their design principles especially activity-based probes and imaging agents are directly applicable to cathepsin biology. Real‑time mapping of cathepsin activity has become feasible through the use of quenched fluorogenic probes and positron‑emission tracers that unmask only after enzymatic cleavage. Joyce *et al*. demonstrated that acyloxymethyl ketone-based fluorescent probes selectively identify cathepsin B-enriched tumor margins *in vivo*, offering high spatial resolution during surgical resection [1]. Subsequent work refined the chemistry to achieve sub‑nanomolar sensitivity and multiplexed read‑outs for cathepsins L and S [139, 140]. Activity‑based probes have also been adapted for immuno‑PET, allowing whole‑body quantification of cathepsin signalling during therapy [141]. For bedside translation, point‑of‑care microfluidic chips now detect cathepsin‑released fluorophores in patient sera within 15 minutes, achieving 90% specificity for metastatic disease [142]. These tools convert cathepsin kinetics into actionable biomarkers for early diagnosis, treatment selection, and response monitoring.

## 9 THERAPEUTIC DELIVERY PLATFORMS EXPLOITING CATHEPSIN CLEAVAGE

Recent therapeutic platforms offer opportunities for cathepsin-selective targeting, especially with stimuli-responsive nanocarriers and linker chemistries tailored for cathepsin cleavage. Several smart carriers now harness the abundant cathepsin activity of diseased tissue to trigger drug release precisely where it is needed. Cathepsin-responsive nanogels cross-linked by Val‑Cit dipeptides remain inert in circulation but disintegrate inside tumour lysosomes, unloading doxorubicin with a ten-fold boost in intratumoural concentration while sparing heart tissue [143]. Antibody–drug conjugates employing the same Val‑Cit linker reach picomolar potency. In a study by Caculitan *et al*., it was shown that cathepsin B-mediated cleavage not extracellular pH dictates the site-specific release of the cytotoxic payload from antibody–drug conjugates, establishing cathepsin B as a molecular gatekeeper in lysosomal drug activation [144]. Liposomes coated with a cathepsin‑K‑cleavable PEG shield penetrate bone metastases, where local K activity removes the steric barrier and accelerates docetaxel release. Complementary efforts pair cathepsin‑S‑degradable polymers with checkpoint inhibitors, providing spatiotemporal control of immunotherapy and reducing systemic cytokine surge [20]. Together, these strategies convert cathepsins from by‑standers into molecular switches that sharpen therapeutic indices and may overcome dose-limiting toxicities of traditional delivery systems.

## 10 FUTURE PERSPECTIVES

### 10.1 Future Challenges in Cathepsin Research

Cathepsin biology has become a rapidly advancing research area, marked by significant progress in both diagnostic technologies and therapeutic development [87, 145]. This section highlights key future priorities, including unresolved technical hurdles, novel therapeutic targets, and strategies to guide clinical translation.

### 10.2 Research Priorities

Despite notable advancements, several critical gaps remain in our understanding of cathepsin function across disease pathways. These include the precise contributions of individual cathepsin isoforms to the progression of diseases, such as cancer, cardiovascular disorders, neurodegeneration, and autoimmune conditions [146, 147]. A comprehensive mapping of isoform-specific roles is essential to uncover disease-relevant mechanisms of action and identifying a new therapeutic window.

For example, the regulatory influence of cathepsins on the tumor microenvironment and their involvement in immune modulation during cancer and chronic inflammation remain incompletely characterized [148]. Moreover, the interplay between cathepsins and other protease systems particularly matrix metalloproteinases (MMPs) has only been partially examined. This interaction may be central to pathological events such as metastatic spread and fibrotic tissue remodeling. Clarifying these molecular relationships could significantly improve the precision and efficacy of targeted interventions [148].

### 10.3 Technical Challenges

However, significant technical challenges remain, for example, how to ensure specificity and selectivity for targeting cathepsins in therapeutic interventions [149]. All cathepsins expand their conserved active sites; this is why it has been difficult to design inhibitors that target only one specific cathepsin without affecting the others, and vice versa. Such a challenge requires advanced strategies in drug design, including the use of allosteric inhibitors or monoclonal antibodies [144].

Current detection methodologies, including ABPs and imaging techniques, also require further development for higher specificity and sensitivity. This has remained a critical challenge: the real-time monitoring of cathepsin activity *in vivo* within complex biological systems [53]. This challenge requires an innovation in molecular probes by the development of imaging techniques that enable the visualization of cathepsins within specific cellular compartments [150].

Cathepsins play a role in the infectious diseases caused by several pathogens that need these proteases to enter and survive in their hosts [82]. Specific inhibitors against cathepsin L, for example, have been shown to mediate the entry of SARS-CoV-2 and thus reduce viral replication [151, 152]. Cathepsin inhibitors will be further researched for parasitic diseases such as malaria and schistosomiasis [82].

Cathepsins are critical for the process of tissue remodeling and extracellular matrix turnover in fibrotic diseases [153]. Broad-spectrum cathepsin inhibitors, such as VBY-825, are being evaluated in early-phase trials for the reduction of fibrosis [134]. The role of cathepsins in fibrotic diseases and tissue remodeling is demonstrated in Fig. (**5**).

Recent investigations extend the novel use of cathepsins beyond the domain of therapeutics. Nanotechnology can now offer the preparation of drug delivery systems reliant on cathepsin-sensitive linkers for the activation of chemotherapeutic agents [154]. The basis underlying nanocarrier drug-delivery methods depends on intratumor enzymatic catalysis, which significantly improves tumor targeting while minimizing off-target toxicities. In the emerging area of regenerative medicine, the action of cathepsins plays an important part in tissue repair. Therapeutic manipulation may accelerate the healing course following myocardial infarction using interference in cathepsin activities [155].

Adaptive trial designs should be foremost as cathepsin inhibitors enter and progress through clinical trials. Such designs will enable modifications to interim results, thereby aiding in the assessment of a new therapy's efficacy and safety [140]. Likewise, strong preclinical models should be integrated into clinical trial design to ensure proper translation from bench to bedside. Biomarker-driven stratification of patients in trials is also crucial for determining which patients will most benefit from therapy [11, 76].

Patient stratification is a crucial aspect of clinical development. It describes the identification of patients based on certain biomarkers, including specific cathepsin expression profiles. In cancers, for example, heterogeneity in cathepsin expression may impact therapy response. Personalization of treatments through molecular profiling can facilitate improved therapeutic efficacy and reduce adverse effects [130].

For clinical application, there is a need for the development of biomarkers of cathepsin activity and disease progression. Biomarkers of cathepsin activity can include circulating levels of the enzyme or specific substrates that would inform on disease dynamics and treatment response [130]. Of course, these biomarkers will enable real-time monitoring and permit timely adjustments in therapy.

Approval from regulatory agencies for therapies targeted at cathepsin is considered challenging due to safety, efficacy, and long-term effect concerns [65]. In this respect, regulatory agencies require comprehensive data from well-designed clinical trials before approving therapies. Researchers must interact with regulatory agencies early in the process to address these challenges and ensure that therapies meet both safety and efficacy standards [77, 110, 113]. Cathepsin research stands at the threshold of breakthroughs that promise to revolutionize therapies across cancer, cardiovascular disorders, neurodegenerative diseases, and fibrosis [83]. Therapeutic success, however, will need to overcome major challenges in selectivity, specificity, and the multifunctional biological roles of cathepsins [22, 99, 156]. Innovation in drug design, improved methodologies for detection, and patient stratification strategies will be the primary avenues through which successful therapies targeting cathepsins can be developed. Interdisciplinary collaboration and the integration of recent innovative technologies are essential to realizing the full potential of this field [56, 157-160].

## CONCLUSION

Cathepsins play critical roles in both disease and healthy tissue processes, with established roles in several diseases, including cancer, cardiovascular diseases, neurodegenerative diseases, and immune system deregulation. Despite significant advances in defining their roles, the contribution of individual cathepsin isoforms to disease progression and inhibition remains poorly understood. In addition, the concomitant presence of both structural homology and redundancy in function creates a significant challenge in the development of selective drugs, which is a significant barrier to therapeutic development.

However, new approaches, including small-molecule drugs, monoclonal antibody therapies, and peptidyl compounds, have facilitated the discovery of early candidates in both clinical and preclinical settings. Breakthroughs in allosteric inhibition, rational design methodologies, and nanotechnology delivery platforms have revealed new avenues for enhancing selectivity and minimizing off-target activity. In addition, the development of reliable markers and real-time imaging tools will become increasingly important for selecting candidates and optimizing therapeutic regimens.

The future will rely on multidisciplinary collaboration between structural biologists, medicinal chemists, and clinicians to develop complex approaches for modulating cathepsins. Translating cathepsin-targeted strategies into the clinic will require harmonizing molecular insight with innovative delivery systems, enabling earlier detection, more precise diagnosis, and real-time monitoring of disease progression.

## Figures and Tables

**Fig. (1):**
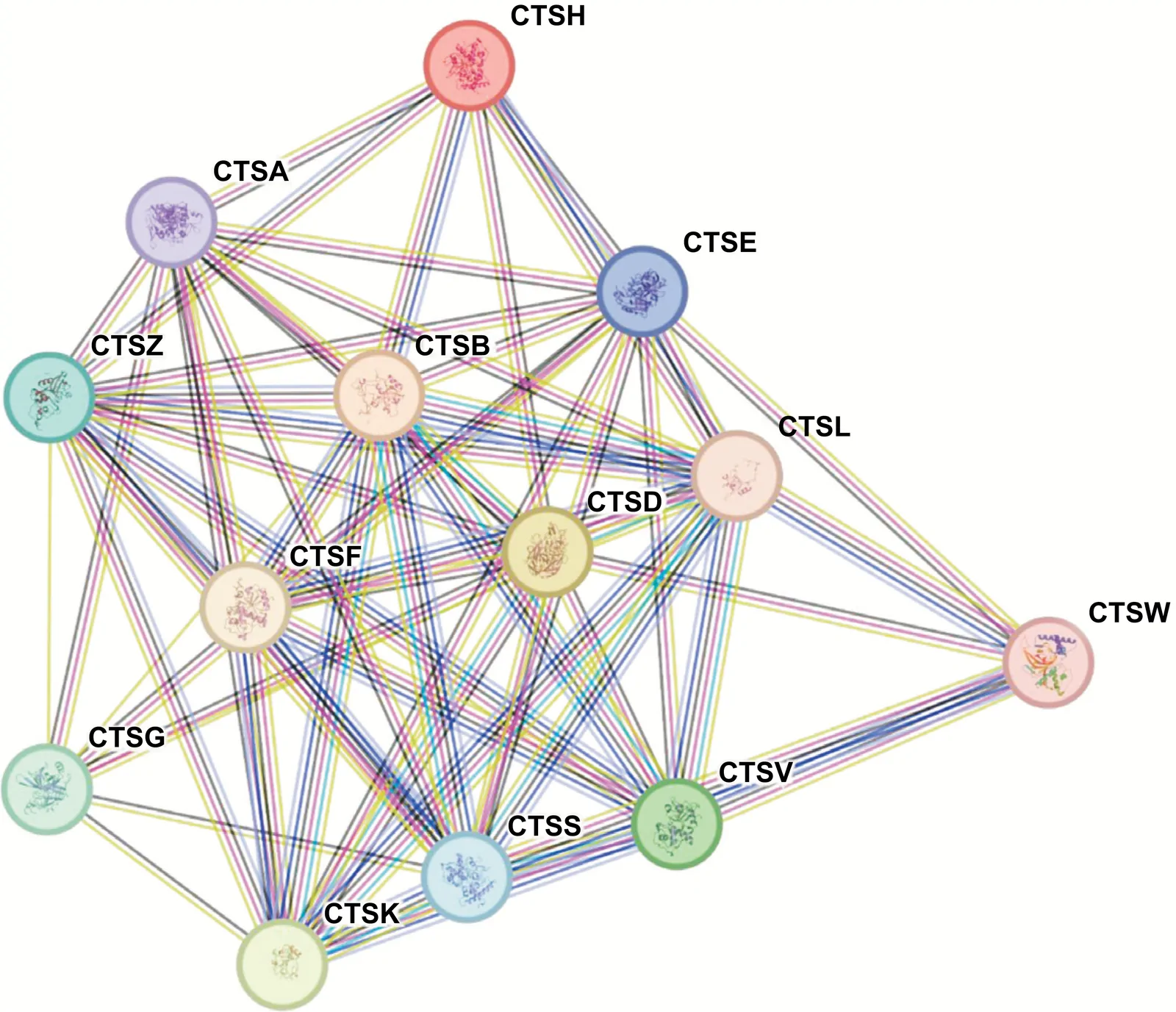
Protein-Protein Interaction (PPI) Network of Human Cathepsins classified by enzymatic type. Node colors indicate protease class (red = cysteine, blue = aspartic, green = serine); edge colors and thickness represent interaction evidence and confidence. Constructed using STRING v11.5 and visualized in Cytoscape 3.10.

**Fig. (2):**
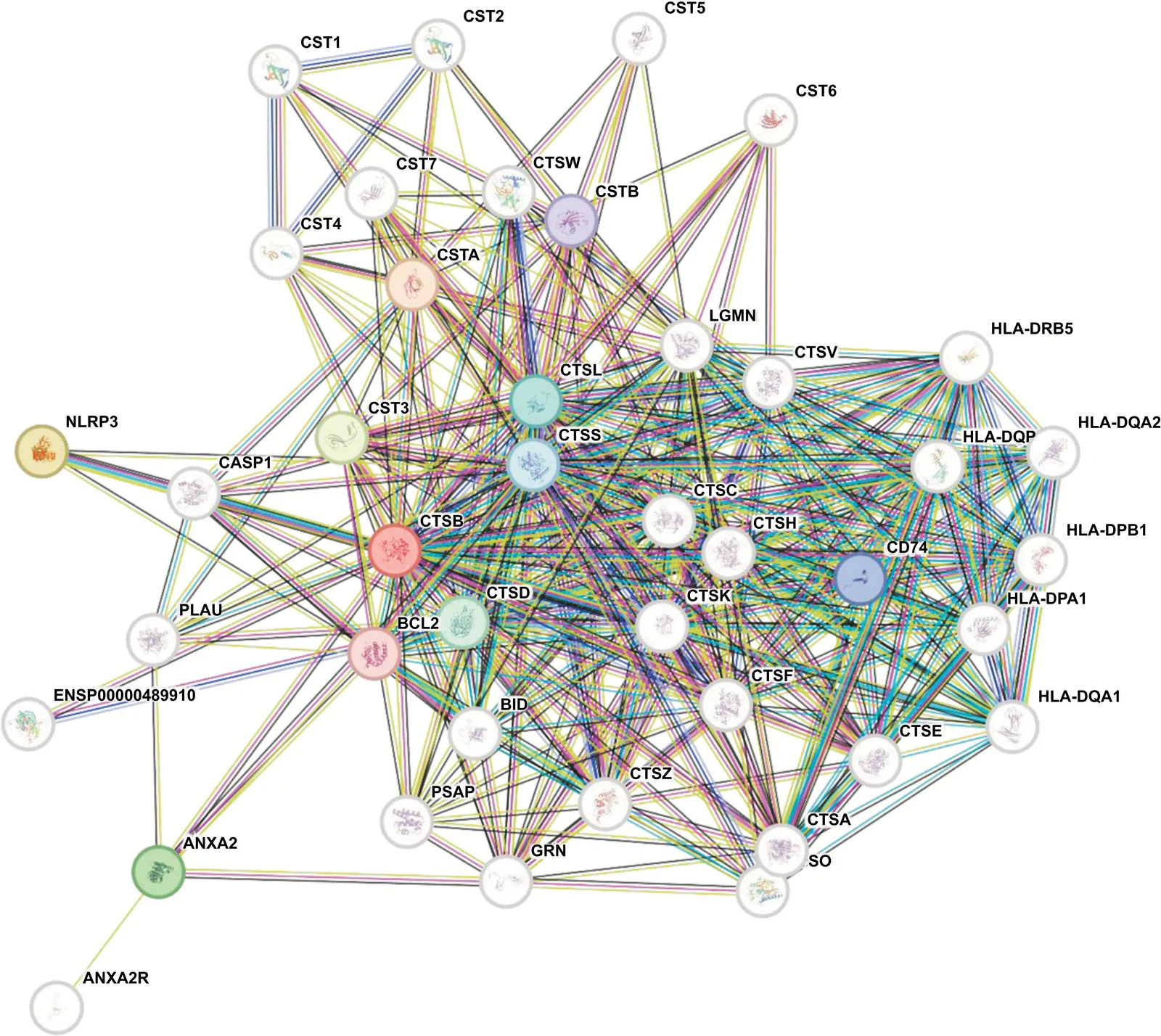
Network depiction of disease-relevant cathepsins involved in cardiovascular and neurodegenerative pathologies, showing functional associations and interaction strength among isoforms implicated in matrix degradation, inflammation, and neuronal damage. Constructed using STRING v11.5 and visualized in Cytoscape 3.10.

**Fig. (3):**
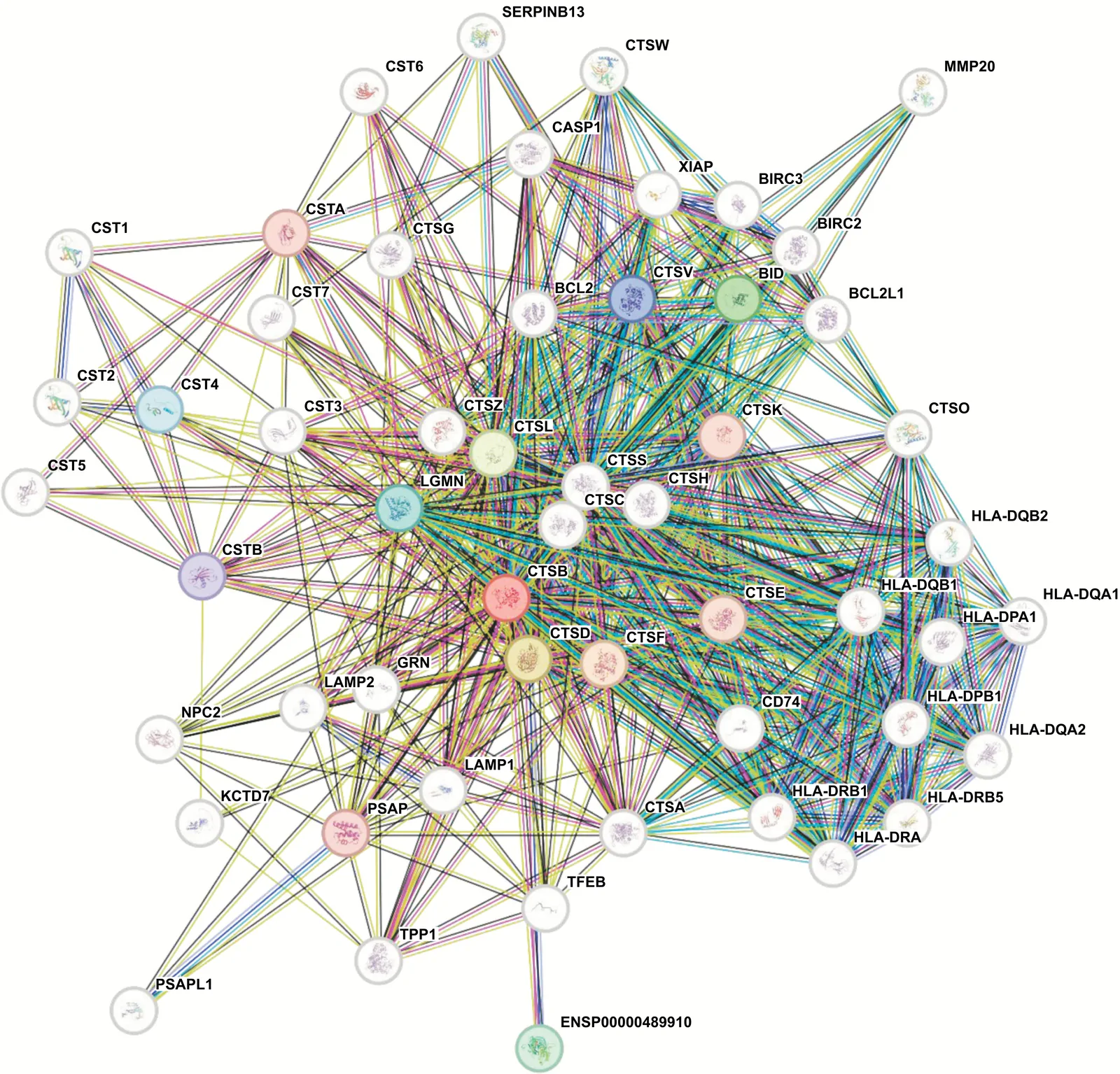
Functional interaction map of lysosomal cathepsins (*e.g*., B, D, L) highlighting their roles in apoptotic and autophagic signaling cascades. Constructed using STRING v11.5 and visualized in Cytoscape 3.10.

**Fig. (4):**
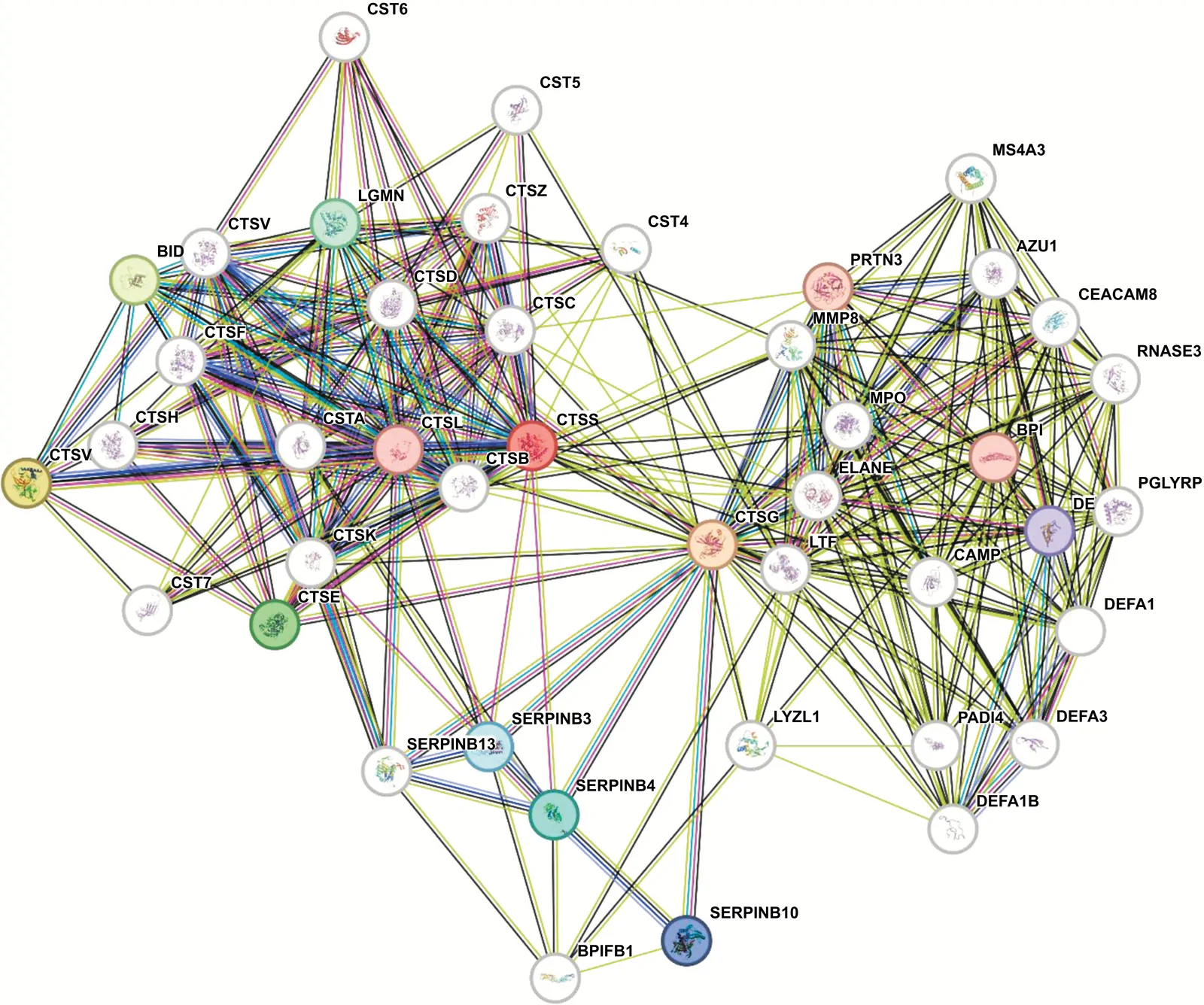
PPI network showing cathepsins involved in immune regulation and inflammation, including antigen processing (cathepsin S), neutrophil activation (cathepsin C), and cytokine modulation (cathepsins L and B). Constructed using STRING v11.5 and visualized in Cytoscape 3.10.

**Fig. (5):**
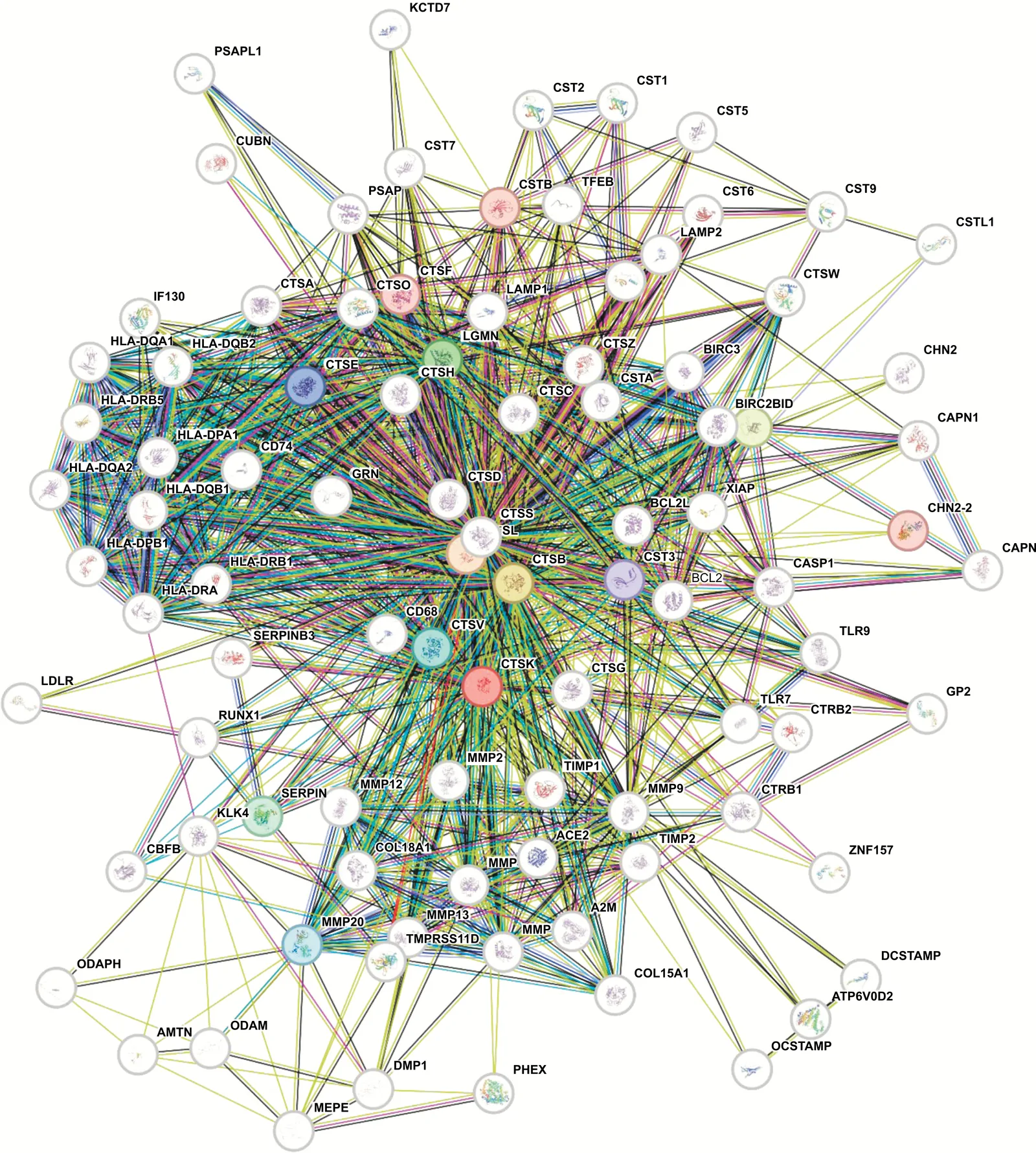
Visualization of cathepsin-driven extracellular matrix remodeling in fibrotic diseases. The network highlights the roles of cathepsins K, L, and S in collagen degradation and fibroblast activation pathways, constructed using STRING interaction data.

**Table 1:** Key human cathepsins: Classification, localization, disease involvement, and therapeutic potential.

**Name**	**Type**	**Function**	**Localization**	**Diseases Involved**	**Therapeutic Applications**
Cathepsin B	Cysteine Protease	Protein degradation and apoptosis regulation	Ubiquitous, especially in the liver and kidneys	Tumor progression, metastasis, neurodegeneration (*e.g*., Alzheimer’s)	Cancer therapies, neurodegenerative disease management
Cathepsin K	Cysteine Protease	Bone resorption, ECM remodeling	Osteoclasts, bone tissues	Osteoporosis, arthritis, and bone metastases	Inhibitors for osteoporosis, cancer therapy
Cathepsin S	Cysteine Protease	Antigen processing, immune response regulation	Immune cells (macrophages, dendritic cells)	Atherosclerosis, autoimmune diseases	Inhibitors for cardiovascular and autoimmune disorders
Cathepsin D	Aspartic Protease	Protein degradation, apoptosis induction	Ubiquitous, especially in the heart and brain	Cancer, neurodegeneration, and cardiovascular diseases	Targeting cancer, neurodegenerative diseases
Cathepsin L	Cysteine Protease	Antigen presentation, immune regulation	Immune cells, lymphatic tissues	Cancer invasiveness, neurodegenerative diseases	Targeting cancer metastasis and inflammatory diseases

**Note**: This table classifies human cathepsins based on catalytic mechanism, subtypes, and subcellular localization.

**Abbreviations**: ECM = extracellular matrix; CNS = central nervous system; MHC = major histocompatibility complex.

**Table 2:** Disease-specific roles of cathepsins: Mechanisms and clinical relevance.

**Cathepsin**	**Active-Site Residue**	**Primary Physiological Roles**	**Tissue Distribution**	**Pathological Implications**	**Therapeutic Targeting and Challenges**
Cathepsin B	Cysteine	Intracellular protein degradation, apoptosis regulation, and ECM remodeling	Ubiquitous, particularly in the liver and kidneys	Tumor progression, metastasis, neurodegeneration (*e.g*., Alzheimer’s), and liver fibrosis	Promising cancer target; off-target effects and dual roles in apoptosis complicate development
Cathepsin L	Cysteine	Lysosomal turnover, antigen processing, pro-hormone activation	High in spleen, thymus, and lymph nodes	Cancer invasiveness, neurodegeneration (*e.g*., tauopathies), skin disorders	Selective inhibitors show promise; balancing physiological and pathological effects is challenging
Cathepsin K	Cysteine	Osteoclast-mediated bone resorption	Bone tissues (osteoclasts)	Osteoporosis, arthritis, bone metastases	Odanacatib reduces resorption; safety concerns hinder clinical progress
Cathepsin S	Cysteine	Antigen presentation, ECM degradation, and immune regulation	Macrophages, dendritic cells, and B cells	Atherosclerosis, autoimmune diseases (*e.g*., SLE), chronic inflammation	Inhibitors like LY3000328 show promise; specificity is needed to reduce side effects
Cathepsin D	Aspartic	Protein degradation, apoptosis induction	Ubiquitous; high in heart, brain, and muscle	Cancer (pro-apoptotic and pro-invasive), neurodegeneration, cardiovascular disease	Dual role complicates targeting in cancer and neurodegeneration
Cathepsin E	Aspartic	Protein degradation, antigen processing	GI tract, immune cells	GI disorders, immune dysfunction	Therapeutic potential in immune diseases remains underexplored
Cathepsin G	Serine	Antimicrobial activity, NET formation	Neutrophils, immune cells	Inflammatory diseases, neutrophil-mediated tissue damage	Requires careful modulation to avoid triggering inflammation
Cathepsin F	Cysteine	Lysosomal proteolysis	Spleen, thymus, lungs	Genetic disorders (*e.g*., cystinosis), immune dysregulation	Little explored: potential therapeutic avenues in immunology
Cathepsin H	Cysteine	Aminopeptidase activity, lysosomal proteolysis	Kidney, liver	Cancer, neurodegeneration	Broad physiological roles limit therapeutic focus
Cathepsin V	Cysteine	Antigen processing, ECM remodeling	Thymus, cornea	Cancer, immune-related disorders	Oncology target under study; selectivity remains critical
Cathepsin X	Cysteine	Cell adhesion, immune signaling	Immune cells, neurons	Neuroinflammation, tumor progression	Early-stage target for neurodegenerative diseases
Cathepsin W	Cysteine	Cytotoxic T-cell function, immune regulation	Cytotoxic T cells, NK cells	Immune dysfunction, viral infections	Emerging immunotherapy target; limited *in vivo* data
Cathepsin A	Serine	Lysosomal degradation, peptide activation	Heart, kidney	Cardiomyopathy, lysosomal storage diseases	Therapeutic use is emerging in lysosomal disorders
Cathepsin Z	Cysteine	Cell adhesion, migration	Brain, immune tissues	Neurodegeneration, cancer	Therapeutic research remains preliminary

**Note**: This table provides an expanded overview of the catalytic class, physiological role, tissue distribution, and pathological relevance of human cathepsins, highlighting the challenges in targeting them for therapeutic purposes. Abbreviations: ECM = extracellular matrix; NET = neutrophil extracellular trap; GI = gastrointestinal; NK = natural killer; SLE = systemic lupus erythematosus.

**Table 3:** Overview of cathepsin-targeting inhibitors: Modalities, therapeutic indications, and development challenges.

**Cathepsin Target**	**Inhibitor Type**	**Example Inhibitors**	**Therapeutic Target**	**Key Challenges**
Cathepsin B	Small Molecule Inhibitors	CA-074, Nitroxoline	Cancer, neurodegenerative diseases	Off-target effects; insufficient selectivity
Cathepsin K	Small Molecule Inhibitors	Odanacatib, Balicatib	Osteoporosis, bone metastases	Cardiovascular side effects: narrow therapeutic window
Cathepsin S	Small Molecule Inhibitors	LY3000328, VBY-825	Autoimmune, cardiovascular diseases	Specificity limitations: inflammatory off-targets
Cathepsin L	Peptide-Based Inhibitors	S-PI, Z-FY-CO₂Et	Cancer, inflammatory diseases	Low stability, poor bioavailability, and delivery
Cathepsin G	Monoclonal Antibodies	Brensocatib	Chronic inflammatory and pulmonary diseases	Immune response activation; antigenic specificity

**Note:** His table highlights prominent inhibitors targeting cathepsins, organized by inhibitor class, with examples, therapeutic applications, and key limitations in clinical development.

**Abbreviations**: S-PI = synthetic peptide inhibitor; CO_2_Et = carboxyethyl ester.

## LIST OF ABBREVIATIONS

ABP: Activity-based Probe
AD: Alzheimer’s Disease
APP: Amyloid Precursor Protein
BBB: Blood-brain Barrier
BMM: Bone Matrix Mineralization
CA-074: Cathepsin B Inhibitor
CD: Cluster of Differentiation
CFU: Colony Forming Unit
CNS: Central Nervous System
COPD: Chronic Obstructive Pulmonary Disease
CTL: Cytotoxic T Lymphocyte
DAMPs: Damage-associated Molecular Patterns
ECM: Extracellular Matrix
ELISA: Enzyme-linked Immunosorbent Assay
GI: Gastrointestinal
H&E: Hematoxylin and Eosin
IL: Interleukin
LC3: Microtubule-associated Protein 1A/1B-light Chain 3
Lys: Lysosome
MAPK: Mitogen-activated Protein Kinase
MHC: Major Histocompatibility Complex
MMP: Matrix Metalloproteinase
NK: Natural Killer (cells)
NET: Neutrophil Extracellular Trap
PAMPs: Pathogen-associated Molecular Patterns
PET: Positron Emission Tomography
PPI: Protein-protein Interaction
PRR: Pattern Recognition Receptor
ROS: Reactive Oxygen Species
SLE: Systemic Lupus Erythematosus
S-PI: Synthetic Peptide Inhibitor
TGF-β: Transforming Growth Factor Beta
TLR: Toll-like Receptor
TNF-α: Tumor Necrosis Factor Alpha
VEGF: Vascular Endothelial Growth Factor
Z-FY-CO2Et: Benzyloxycarbonyl-phenylalanyl-tyrosyl-carboxyethyl ester
